# Hypoxic ischemic encephalopathy (HIE)

**DOI:** 10.3389/fneur.2024.1389703

**Published:** 2024-07-23

**Authors:** E. Cuauhtémoc Sánchez-Rodríguez, Vasthi J. López

**Affiliations:** ^1^Department of Hyperbaric Medicine, General Hospital Agustin O'Horan, Servicios de Salud de Yucatan (SSY), Mérida, Yucatán, Mexico; ^2^Department of Hyperbaric Medicine, Faculty of Medicine of National Autonomous University of México (UNAM), Mexico City, Mexico; ^3^Department of Hyperbaric Medicine, Naval Academy of Mexico (Armada de Mexico), Mexico City, Mexico; ^4^Institute of Global Health, Michigan State University (MSU), East Lansing, MI, United States; ^5^AMINOXIALAB Laboratory, Department of Biomedical Sciences, Faculty of Medicine, Catholic University of the Northern Chile (UCN), La Serena, Chile

**Keywords:** acute ischemic hypoxic encephalopathy, hyperbaric oxygenation therapy, mitochondria, ischemia-reperfusion injury, antioxidant effect

## Abstract

**Introduction:**

The morbidity and mortality of acute ischemic hypoxic encephalopathy in newborns have not been dramatically modified over the last 20 years. The purpose of this review is to describe the use of hyperbaric oxygenation therapy (HBOT) in the management of acute ischemic hypoxic encephalopathy in newborns.

**Methods:**

A review of the medical literature was conducted on the use of HBOT in the pathophysiology of this condition and its impact on outcomes of patients treated at an early stage.

**Results:**

When HBOT is administered promptly, it can promote the survival of the penumbra, modulate the cytokine storm, modify inflammatory cascades, restore mitochondrial function, inhibit apoptosis, reinstate cellular communication and cytoskeleton function, reinstall the functioning of the kinase system, reduce cytotoxic and tissue edema, promote microcirculation, and provide an antioxidant effect. All these secondary mechanisms aid in saving, rescuing, and protecting the marginal tissue.

**Conclusion:**

When used promptly, HBOT is a non-invasive adjunct treatment that can preserve the marginal tissue affected by ischemia, hypoxia, meet the metabolic needs of the penumbra, reduce inflammatory cascades, prevent the extension of the damaged tissue, and modulate ischemia-reperfusion injury.

## Introduction

Hypoxic ischemic encephalopathies (HIE) carry a high risk of death or disability. Annually, 15 million people worldwide suffer a stroke, with approximately 33% of the cases resulting in death and another 33% leading to permanent disability. Despite all the efforts to reduce morbidity and mortality of stroke, the absolute number of strokes continues to increase. One of the most important factors related to the increase in cases appears to be the longer life expectancy observed over the last 20 years. The incidence of stroke increases significantly in patients older than 65 years (1).

The Global Burden of Disease (GBD) study showed that the absolute number of strokes increased by 70% over 30 years (1990–2019). Globally, there was an increase in prevalent cases of 119%, in disability-adjusted life years (DALYs) of 115%, and in annual deaths of 146% (2).

Hypoxic ischemic encephalopathy (HIE) represents a global hypoxic insult to the brain. This complex neurologic dysfunction is estimated to affect 1.5 per 1,000 term births and accounts for 15–35% of all cases of neonatal encephalopathy in preterm and term infants. It includes three phases, namely, the hypoxic/ischemic insult; mitochondrial dysfunction, excitotoxicity, inflammation, and oxidative stress; and cell death, remodeling, repair, and gliosis (3).

## Pathophysiology of hypoxic ischemic encephalopathy (HIE)

Life on Earth has evolved in an oxygen-rich atmosphere, and oxygen is essential for breathing, cellular metabolism, cell signaling and communication, inflammation, oxidative damage, and cell death. Many of these processes are mediated by reactive oxygen species (ROS) (4–6).

During hypoxia, oxygen is not available in sufficient amounts at the cellular and tissue levels. The inadequate delivery of blood supply and/or the low oxygen content in the blood will compromise homeostasis. The central core of the lesion will undergo necrosis, while the marginal tissue will suffer from an ischemic and metabolic penumbra. Both penumbras may affect the glial cells and neurons, especially those in the hypothalamus, which are key components in regulating energy homeostasis (7).

Ischemia-reperfusion injury (IRI) is the damage caused by the restoration of blood supply to the tissues (reperfusion/reoxygenation) after a period of ischemia and hypoxia. IRI often results in more tissue damage than the initial ischemic insult. Research has focused on identifying the cellular pathways involved in causing damage to organs by IRI. It is associated with a reduced production of ATP at the ATPase-synthase level, with the concomitant increase in the production of reactive oxygen species (ROS) within the mitochondria (7, 8).

Hypoxia-inducible factor-1α (HIF-1α) is an oxygen-sensitive transcription factor that plays a crucial role in regulating and mediating adaptative metabolic responses to hypoxia and cerebral ischemia. It regulates more than 100 genes and participates in many processes, including metabolism, proliferation, preconditioning, postconditioning, activation of signaling pathways, and angiogenesis. HIF-1α also regulates PI3K, VEGF, EPO, MKP-1, Glut-1, and many glycolytic enzymes, including glyceraldehyde 3-phosphate dehydrogenase, phosphoglycerate kinase 1, 12-lipoxygenase, proapoptotic members of the Bcl-2 family (Bnip3), and carbonic anhydrase (9–13).

During hypoxia, mitochondrial ROS production is associated with an increased expression of HIF-1α. The genes that are expressed downstream of HIF activity enhance oxygen-independent ATP generation, cell survival, and angiogenesis. Therefore, they are important factors for tissue protection during IRI. Studies performed in different cell types have shown that HIF-1α regulates hypoxic upregulation of genes, including cyclin D1, transforming growth factor α, POU5F1, and matrix metalloproteinase 2 (MMP-2) (9–13). Therefore, HIFs are rapidly stabilized upon the loss of the oxygen supply, resulting in an orchestrated transcriptional response to modulate cellular phenotypes. This transcriptional response has wide-ranging beneficial effects during the reperfusion of tissues (14).

HIF-1α and HIF-2α share 48% identity. HIF-2α is constitutively expressed in the brain but is not induced by 3 h of hypoxia in the neonatal rat brain. It is expressed mainly in the glial and endothelial cells during normoxia and in less severe hypoxia. The loss of neuronal HIF-2α exacerbated brain injury in the acute (< 24 h) and subacute (< 6 h) phases of HIE, with a trend toward more severe volume loss in the adult brain. HIF-2α also regulates antioxidant genes, angiogenesis, and cerebral microvasculature reconstruction during brain recovery, playing a beneficial role in maintaining reactive oxygen species and mitochondrial homeostasis (15).

The blood–brain barrier (BBB) is composed of microvascular endothelial cells, astrocytes, neurons, pericytes, and the basement membrane. Oxidative stress during ischemia affects the interaction between endothelium cells and pericytes, leading to blood flow reduction and BBB breakdown. Pericytes appear to be more sensitive to ischemic injury than endothelial cells. HIF-1α inhibition decreases BBB damage by regulating matrix metalloproteinase-2 (MMP-2) and vascular endothelial growth factor (VEGF). The loss of HIF-1 in pericytes reduces ischemia-induced pericyte death, subsequently reducing BBB permeability and CNS transendothelial leakage (16, 17).

HIF-1α could serve a dual role in cell survival or death during cerebral ischemia/hypoxia. It might induce cell death during severe and long-term ischemia/hypoxia but could promote cell survival under mild ischemic stress (9). In the early stages of ischemia, the inhibition of HIF-1α lowers brain injury, edema, and apoptosis. However, in the recovery phase, neuroprotective effects might be achieved by promoting angiogenesis through the induction of VEGF expression (8, 18).

## Ischemic reperfusion injury (IRI)

Hypoxic ischemic encephalopathy (HIE) not only generates an ischemic and metabolic penumbra but also creates an ischemia-reperfusion injury (IRI). The impact of ischemia/hypoxia is not uniform across the brain, with several local and systemic factors determining the magnitude of the damage and its potential reversibility. Two key factors involved in the reversibility of the injury are ATP levels and time. Once the mitochondrial oxidative phosphorylation system (OXPHOS) is compromised, a series of detrimental events occur, often simultaneously and/or sequentially (18).

Mitochondria are responsible for ATP production, metabolism, cell signaling, and energetic regulation. An energetic balance is needed to meet the energy needs during normal and stressful conditions. The brain is particularly vulnerable to hypoxia because it consumes 20 times more ATP (4.7 x 10^9^) than the rest of the body (19). Mitochondria also chelates calcium (Ca^++^) from the cytosol when ion pumps fail. It is also responsible for producing most of the reactive oxygen species (ROS), cell-cell communication, intrinsic apoptosis and maintaining the redox balance. It is also the only organelle that has DNA and shares 30% of its DNA with the nucleus. It is theorized that this is an adaptative cell process to maintain adequate energetic balance, homeostasis, allostasis, and allostatic responses, which are key for enduring stress conditions (20–22).

One of the mechanisms used to survive low oxygen levels is the severe depression of the metabolic rate during oxygen deprivation in association with lower rates of ATP production via the fermentative pathways (the Warburg effect), which become a key strategy for survival (21). The reduction in ATP availability affects membrane ion pumps, the expression of phosphatases, kinases, transcription factors, and microRNAs (22, 23).

The dysfunction of mitochondrial membrane ion pumps affects cellular ion exchange (Na^+^/K^+^, Na^+^/Ca^++^). Sodium (Na^+^) increases in the cytosol and promotes cytotoxic edema. Brain swelling is also associated with the influx of water into the perivascular astrocytes through channels called aquaporins (AQP). Cerebral ischemia promotes the influx of Na^+^ through SUR1-TRPM4-induced Ca^++^ transport into the cells, which increases the calmodulin-dependent translocation of AQP4 to the plasma membrane and water influx into the cell (24, 25).

NA^+^ acts as a second messenger that regulates OXPHOS function, redox signaling, and the production of ROS by modulating the fluidity of the inner mitochondrial membrane. A conformational shift in mitochondrial complex I during acute hypoxia drives acidification of the matrix and releases free Ca^++^ from calcium phosphate precipitates. The activation of the mitochondrial Na^+^/Ca^++^ exchanger promotes the import of Na^+^ into the matrix. This reduces the mobility of free ubiquinone between complexes II and III with the subsequent production of superoxide (O${\;}_{2}^{-}$) in complex III (24).

During hypoxia, a mitochondrial paradox arises. ROS are produced at low oxygen levels. Many observations indicate oxidative stress and/or redox imbalance during low oxygen stress. This is possibly caused by the “electron scape” from the respiratory chain. Complexes I, II, and III are the main mitochondrial sources of O${\;}_{2}^{-}$. It is proposed that several conditions must occur for this paradox. There must be changes in complexes I and III and the participation of NADPH. Moreover, complex II switches its catalytic activity from succinate dehydrogenase to fumarate reductase, creating ROS generation because fumarate reductase is a powerful O${\;}_{2}^{-}$ generator (24).

As hypoxia progresses and mitochondria dysfunction increases with a concomitant reduction of ATP beyond a critical level (< 1 mol/kg), necrosis occurs. In the hypoxic/anoxic state, cytochrome C is separated from the internal membrane, and the transition pore opens with the subsequent release of Ca^++^ into the cytosol. It is then when the intrinsic apoptotic cascades mediated by Caspase 3 become irreversible, and programmed cell death type I (apoptosis) occurs. Moreover, TNF-α promotes apoptosis via the extrinsic pathway by interacting with FAS receptors and ligands on the surface of cells (22, 26).

Ferroptosis is another programmed cell death caused by an imbalance in iron metabolism and lipid peroxidation by ROS. Due to the Fenton reaction, non-transferrin-bound iron (NTBI) generates free radicals, leading to lipid peroxidation and triggering ferroptosis. It is a key mediator of cortical mitochondrial damage, hippocampal neuronal death, and neonatal HIE. Newborns are susceptible to ferroptosis due to their abnormal iron metabolism, decreased activity of antioxidant enzymes, and the accumulation of ROS (18, 19).

Ca^++^ ions also act as second messengers and are one of the primary mediators of inflammation. Once the cell enters this energetic crisis, calcium influx into neurons stimulates the glutamate receptors, which activate de nitric oxide synthase isoforms (nNOS and iNOS), causing brain injury. Nitric oxide (NO) permeates membranes and reaches mitochondria, which react with superoxide (O_2_)^−^ to yield peroxynitrite (ONOO^−^). Excess of intracellular calcium also increases mitochondrial superoxide and hydrogen peroxide formation. High mitochondrial oxidant levels can overwhelm the mitochondrial antioxidant system, promoting the generation of stronger oxidants, such as hydroxyl radicals (^·^OH). NO is also involved in the regulation of hypoxia-related genes and might stabilize hypoxia-inducible factor (HIF), a key component of hypoxic acclimation (22, 23, 26–29).

Calcium also mediates other inflammatory cascades. It activates calcium-dependent proteases, which mediate the conversion of xanthine dehydrogenase to xanthine oxidase, enhancing the production of ROS. Calcium also stimulates phospholipase-2 with the subsequent elevation of cyclooxygenase (COX), lipoxygenase, leukotriene, thromboxane, and prostaglandins. Another cascade is the cytokine cascade, mediated by nuclear transition factor kappa B (NFkB). It mediates approximately 150 different cytokines, both inflammatory and anti-inflammatory. It is not well understood how NFkB promotes inflammatory or anti-inflammatory cytokines, but it might involve modulating the redox balance of the cell. It also promotes the production of endothelins, chemokines, interferon, transcription factors, metalloproteases, heat shock proteins, glutamate, caspases, HIF-1α, and NO (22).

The endothelium and the extracellular matrix (ECM) integrate many functions and are probably the first alarm system of the body. Ischemia, hypoxia, and hypoglycemia stimulate the expression of intracellular adhesion molecules (selectins, VCAM, and ICAM-1), the neutrophil integrin-β2, and the release of signaling molecules (cytokines, endothelins, chemokines, transcription factors, kinases, and growth factors) (11). An important reduction in the bioavailability of NO is one of the most important factors in endothelial dysfunction. Oxidative stress leads to eNOS uncoupling and promotes the production of superoxide instead of NO (30).

Endothelial cells possess mechanical, shear stress, and biochemical sensors. The structural and biochemical changes in the endothelial cells contribute to neuroinflammation (31). The blood flow is reduced during ischemia, which, in turn, reduces shear stress, stimulating changes in the actin network of the endothelial cells and resulting in the formation of stress fibers (32). Ischemia also induces changes in F- and G-actin, leading to the reorganization of the cytoskeleton (33). The changes in the cytoskeleton also affect the cell-cell junctions, especially the tight and gap junctions, which are significantly affected by hypoxia. The blood–brain barrier (BBB), an endothelial system, is particularly prone to dysfunction during hypoxia.

There is also a crosstalk between cellular geometry and TNF-α signaling. TNF-α induces geometry-dependent actin depolymerization, enhancing IkB degradation, NFkB translocation, and a geometry-dependent-gene expression pattern (34). These observations confirm the participation of tensegrity during ischemia and hypoxia, where form creates a function and function generates form.

The plasma membrane allows the cell to sense and adapt to changes in the extracellular environment. One of the critical cellular signaling pathways involved in this adaption is the Hippo pathway. This pathway acts as a nexus and integrator of cellular responses to tension, stretching, and changes in the extracellular matrix (ECM) properties. The activation of the Hippo pathway and transcriptional changes through the MST/YAP/FoxO pathways can lead to apoptosis (35). Several components of the Hippo pathway, including YAP, TAZ, KIBRA, LATS1/2, and MST1/2, can temporarily localize to junctional complexes, establishing a good interplay between cellular junctions and the Hippo pathway (36). The sensors, effectors, and transcription factors within this pathway respond to various stimuli and adjust to new and critical environmental conditions. During ischemia and hypoxia, numerous stimuli are produced at the plasma membrane. There must be a system that coordinates, synchronizes, and prioritizes these signals. This coordinating system appears to be the kinase system, which includes phosphatases. Two of the most important kinases in hypoxia are AMP-activated protein kinase (AMPK) and serine/threonine kinase (AKT) (37).

Hypoxia is accompanied by nutrient starvation. Hypoxic signaling is closely linked to nutrient signaling. In nutrient signaling, AMPK and the mechanistic target of rapamycin complex-1 (mTORC1) crosstalk to sense cellular ATP, glucose, and amino acid levels. AMP is a more sensitive indicator of cellular energy states than ATP and activates AMPK. AMPK acts by activating catabolic pathways to facilitate ATP generation once its cellular levels start to decrease. AMPK and mTORC1 modulate several cellular responses during stressful conditions, including mitochondrial respiration, ROS production, protein translation, metabolic reprogramming, and programmed cellular death Type II (autophagy) (37, 38).

AKT stimulation leads to temporal phosphorylation profiles in endothelial cells, affecting growth factor signaling, angiogenesis, cellular protection against oxidative stress, and neuronal damage. Growth factors and insulin stimulation lead to the activation of phosphoinositide-3-kinase (PI3K), the principal mediator of autophagia. The recruitment of AKT to the plasma membrane promotes the phosphorylation of several substrates and residues of Thr308 and Ser473 of rapamycin complex 2 (mTORC2), which are essential for AKT activity. AKT signaling plays a significant role in cellular protection against oxidative stress and neuronal damage (39).

Mitochondria can also actively regulate innate immune responses in infections and sterile conditions. It can directly activate the immune response and modulate it. Pathogen-associated molecule patterns (PAMPs) serve to identify organisms as foreign. Damage-associated molecular patterns (DAMPs) are released or modified during mitochondrial damage in hypoxia. They are recognized as alarmins by receptors of the innate immune system and trigger the immune response. It appears that the loss of mitochondrial membrane integrity, resulting in the escape of components into the cytosol, is the driving force behind this response. However, the exact mechanism is not yet well understood. ATP is included in the mitochondrial alarmins when it is expelled extracellularly by apoptotic or necrotic cells and sensed by the P2X7 receptor to trigger innate immune responses, including the NLRP3 inflammasome (39).

The families of TLR are transmembrane proteins that recognize endogenous ligands and participate in inflammation pathways. Mitochondria are implicated in TLR signaling through TNF receptor-associated factor 6 (TRAF6). Mitochondrial gene expression is upregulated downstream of both TLR3 and TLR4 through the activity of the PPAR-y coactivator family. TLR-4 activation during stress-induced hypoxia of neuronal cells induces an inflammatory response through the formation and activation of autophagy and NLRP3 inflammasomes, which induce IL-1ß release. TLR4 knockdown significantly suppresses the expression of TLR and inhibits apoptosis (39, 40).

Besides cytokines, signaling molecules, leukocyte migration, activation, and the complement cascade compose the innate immune system. In HIE, cytokines are central to the propagation of the immune response, particularly IL-1ß. This can result in direct neural injury that culminates in cell death (pyroptosis). Leukocyte chemotaxis and activation are central to the second phase of the cerebral ischemic reperfusion injury (IRI). The activation of the complement cascade facilitates the activation of leukocyte and endothelial cells and an increase in the release of cytokine. Complement participation after cerebral IRI plays a role in the classical, alternative, and lectin pathways (41).

## Animal models of hypoxic ischemic encephalopathy (HIE) and hyperbaric oxygen therapy (HBOT)

Most of the experimental data come from the neonatal hypoxia-ischemia model developed by Rice and Vannucci in rats (42). The animal data show promising applications of HBOT in acute HIE. In Table 1, we summarize the results of animal HIE and HBOT (43–61).

**Table 1:** Use of HBOT in animal models of HIE.

**References**	**Model**	**N**	**Area/Mediator**	**Treatment protocol**	**Results (SA)**	**Comments**
Wei et al. (43)	7-day-old rat pups in 3 groups (sham, HI control, and HI_HBO). Carotid ligature, 2h 8%O_2_	120	Pyramidal cells, glial cells, and hippocampal dentate gyrus	HBO/min/QA	*p* < 0.001, *p* < 0.05	Promoted repair and regeneration of the nervous system and contributed to the self-recovery and protection of damaged brains
Xue et al. (44)	Adult rats. Sham, control, HI, Hi-1.5ATA, and HI-2.5ATA	60	Hippocampus. Prefrontal cortex. IL1ß, IL6, TNFa, HIF1a, and SOD	HBO 1.5–2.5 ATA/60 min/QA for 6 days	*p* < 0.01, *p* < 0.05	Protects myelin injury, promotes differentiation into oligodendrocytes, inhibits neuroinflammation, and balances oxidative damage and antioxidant activity; 2.5 AT A showed better results than 1.5 ATA
Wang et al. (45)	7-day rat pups, carotid ligation/cut + 2 h hypoxia, study 30 days	70	HBO at 3,6,12,24, and 72 h. Stem cell proliferation and behavior evaluation	HBO 2.0 ATA/60 min/QD for 7 days.	*p* < 0.05	Increases proliferation of neural stem cells, performs better in behavior tests, and causes less neural loss in hippocampal CA1; best at < 12 h.
Chen et al. (46)	7-day rat pups, carotid ligation/cut + 2 h hypoxia, HBO at 1 h	80	Brain histopathology 7 days, water maze test 30 days; caspase 3 Nogo-A, +water maze test	HBO 2.5 ATA/120 min/QD for 7 days; w/ephedrine	*p* < 0.01, *p* < 0.05	Reduces Caspase 3, Nogo-A and improves the Morris water maze tests after 4 weeks.
Chen et al. (47)	14-day-old rats, SOD, MDA, VEP	40	Antioxidant, lipid peroxidation, brain synapsis CA3, and P1 VEP	HBO 2.0 ATA/60 min/QD for 14 days.	*p* < 0.01, *p* < 0.05	Enhances antioxidant capacity, less ultrastructural damage, improves synaptic reconstruction, and promotes brain function
Yin et al. (48)	7-day rat pups, BMP-4, HBO at 6 h HIE	30	BMP-4, nestin, tunnel in the hippocampus	HBO 2.0 ATA/40 min/QD for 7 days	*p* < 0.01	Promotes neurological recovery and inhibits neural apoptosis.
Feng et al. (49)	7-day rat pups, BMP-4, nestin, tunnel in the hippocampus HBO at 6 h HIE	108	Brains removed at 1,3,5,7,14,21 days	HBO 3 ATA 7 60 min/QD for 7 days	*p* < 0.05, *p* < 0.01	Proliferates neural stem cells, improves recovery, and increases nestin
Wang et al. (50)	7-day rat pups, Wnt 3, nestin, subventricular zone, HBO at 6 h HIE	150	Stem cells, nestin, WNR-3, at 6,24, and 7h and 7,14 days	at 3 h HIE HBO2 ATA/60 min/QD/	*p* < 0.05, *p* < 0.01	Proliferation/migration of neural stem cells subventricular zone, increased nestin, and Wnt-3
Wei et al. (51)	7-day rat pups, NGF, HBO at 6 h HIE	40	NGF, water maze, sensory-motor function at 30 and 42 days	HBO 2.0 ATA/30 min/QD for 7 days	*p* < 0.01	Improves synaptogenesis, dendritic changes, and synaptic remodeling. Reduces escape latency and piercing index and improves sensory motor function
Yang et al. (52)	7-day rat pups, NGF, HBO at < 1 h HIE	360	Mitochondrial function/MTP at 0,2,4,6, and12 h 2,3,4,5,6, and 7 days	HBO ATA/1 h/QD for 7days	*p* < 0.05	Mitochondrial function/MTP after HBOT single dose at 2,3,4,5,6 and 7 days
Li et al. (53)	7-day rat pups, HBO at 24 h before HIE		Caspase 3,9, TTC, and tunel	7-day rat pups, HBO, and HBO 2.5 ATA/150 min	*p* < 0.05	Preconditioning HIE increases survival and reduces infarction apoptosis
Chen et al. (54)	7-day rat pups, HBO < 24 h	21	Wnt3 -ß-catenin, BMP. NSC to neuron, oligodendro-cytes	HBO 2.0 ATA/60 min	*p* < 0.05	Promotes differentiation of NSC into neurons, oligodendrocytes, increases astrocytes through Wnt-3/ß-catenin, BMP2. HBO 1 h > 30 min, 2 ATA > 3 ATA
Calvert et al. (55)	7-day rat pups, HBO at 1 h	30	Caspase 3 at 18–24 h, PARP at 18–48, Tunel, cortex, and hippocampus, brain removed 12,18,24, and 48 h at 4–6 weeks.	HBO 3 ATA/60 min	*p* < 0.05	Increases neuroprotection, reduces apoptosis and Caspase 3 cortex, hippocampus, reduces DNA fragmentation, tunel, and preserves brain weight,
Calvert et al. (56)	7-day rat pups, HBO at 1 h	144	ATP, ATPase, hippocampus, and cortex at 4,24, and 72 h	HBO 2.5 ATA/120 min	*p* < 0.05	Reduces IRI, brain weight, and morphology, 2.5 ATA > NBO
Calvert et al. (57)	7-day rat pups, HBO at1 h	144	ATP, ATPase, hippocampus + cortex at 4,24, and 72 h	HBO 2.5 ATA/120 min	*p* < 0.05	Reduces IRI, brain-weight and morphology, 2.5 ATA > NBOT
Calvert et al. (58)	7-day rat pups	134	at 1,1.5,3 ATA histology at24h, 1,2, and10 weeks and 2, HIF1∞, and VEGF	HBO1.5-3 ATA/60 min	*p* < 0.05	Protects against retinopathy, retinal vascular density, < brain weight, no > HIF1∞, VEGF
Günther et al. (59)	Hypoxically damage rat neocortical brain slices at 5-30 min hypoxia	-	HPLC, Celestine blue/fuchsin staining, ATP/ADP, GTP/GDP	HBO 2.5 ATA/30–120 min	*p* < 0.05, *p* < 0.01	HBO 2.5 ATA/60 min > NBO at 5 min better than 30 min
Liu et al. (60)	7-day rat pups, HBO at 1 h	108	Neuroprotection at 28–60 days, histology, Caspase3, AIFat 12,24, and 48 h, sensorimotor, Morris water maze test, and tunel	HBO2.5 ATA/90 min/QD	*p* < 0.05, *p* < 0.001	HBO promotes long-term functional and histological recovery, induces neuroprotection, suppresses apoptosis, and inhibits Caspase-3 and AIF pathways
Calvert et al. (61)	7-day rat pups, HBO at 1 h	151	Ipsilateral hemisphere weight, sensorimotor tests at 5 weeks	HBO 3 ATA/60 min	*p* < 0.05	HBO reduces atrophy, and apoptosis and improves sensorimotor function at 5 weeks

## Hyperbaric oxygen therapy (HBOT)

HBOT is a treatment where a patient breathes 100% oxygen inside a pressure vessel designed for human occupancy, either a monoplace or multiplace hyperbaric chamber, at ambient pressures ranging from 1.5 to 3.0 atmosphere absolute (ATA). Each treatment session takes 60–120 min and can be conducted one to three times daily, depending on the medical condition of the patient. Acute pathologies, such as CO poisoning, might require one treatment, and chronic osteomyelitis might take 40 treatments. Only one treatment might be needed in the early management of acute HIE (within 4–6 h after delivery). The subacute cases might require more.

HBOT is based on gas laws, particularly Henry's law. The primary mechanism of HBOT is hyperoxygenation. The plasma partial pressure obtained at 2.0 ATA is close to 1,500 mmHg and close to 2,000 at 3 ATA. This hyperoxygenation effect creates temporary oxidative stress during the first 60 min of treatment, but it also creates an important antioxidant effect that remains for 72 h after the last treatment (62–64).

Hyperoxygenation produces several secondary mechanisms. Hyperoxygenation increases the diffusion of oxygen from the vascular space into the tissular space, restoring ATP production and cellular and tissular oxygen tension and promoting cell survival in the ischemic and metabolic penumbra (62–64).

It also breaks the vicious cycle of edema-hypoxia-edema. The edema-reduction effect is also caused by arterial vasoconstriction. It depends on the partial pressure of oxygen and the tissue involved. Vasoconstriction is more pronounced in the central nervous system (65). HBOT enhances K-ATPase activity, increases active NA^+^ transport, and accelerates edema clearance. It also reduces endothelial damage and restores cell-to-cell junctions (22, 24). HBOT reduces the mRNA and protein expression of aquaporin (66). The reduction of tissue edema improves cerebral microcirculation.

HBOT facilitates the correction of mitochondrial dysfunction, improves the integrity of compromised mitochondrial membranes, and inhibits secondary cell death by causing the transfer of mitochondria from astrocytes to neurons. It also upregulates ATP expression through increased NAD^+^ expression, an important marker of energy metabolism. There is an upregulation of the Sirt1 expression with a concomitant reduction of the expression of p53 and NFkB. The restoration of the mitochondrial transmembrane potential by HBOT is associated with a significant reduction of the intrinsic apoptotic cascade mediated by caspase 3 and caspase 9 and an increased expression of Bcl-2 and Bcl-xL that inhibit apoptosis. There were no significant changes in the levels of the proapoptotic protein Bax, as Bcl-2 inhibits it (66–68).

**Figure 1:**
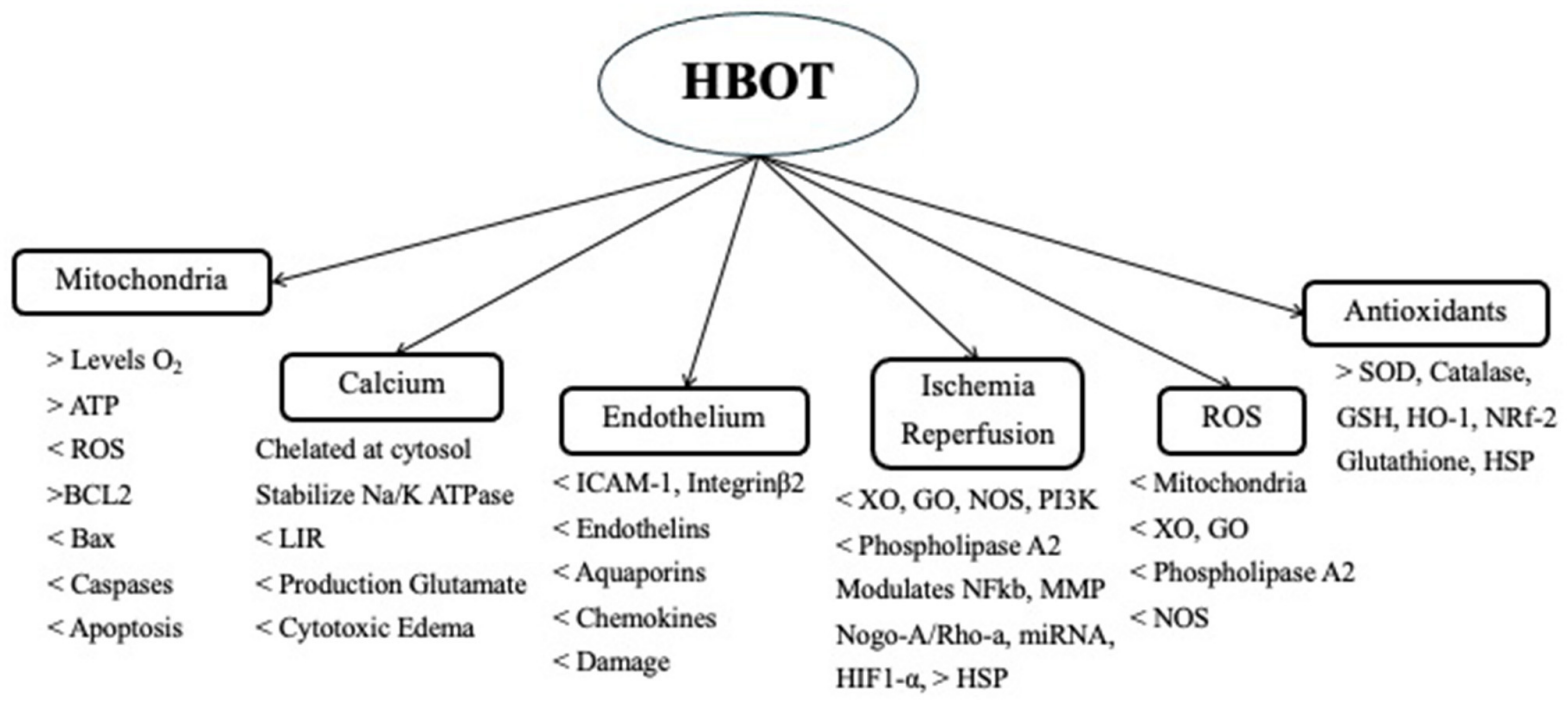
Use of HBOT in IRI.

HBOT has also been shown to reduce calcium overload, which is associated with a reduction in the intracellular calcium level and the inhibition of autophagy through decreased expression of p53 mRNA, AMPK, and mTOR (40). HBOT attenuates the increase in IRI and HIE of IL-1ß, IL-6, IL-8, INF-Y, TNF-α, HIF-1α, ICAM-1, and Integrin-ß2 (*p* < 0.05) (65, 69, 70). It has also shown protective effects by increasing the levels of eNOS. HBOT attenuates neuroinflammation not only by reducing the secretion of proinflammatory cytokines but also by increasing the expression levels of the anti-inflammatory cytokines IL-4 and IL-10 (66–70). HBOT modulates neuroinflammation by decreasing the expression of CCL2/CCR2, matrix metalloproteinase-9, and TNF-α. It also inhibits secondary brain injury by activating the TLR4/NFkB, JNK, p38-MAPK-CCR2, and ERK signaling pathways (45). The effects of HBOT on HIE and IRI are shown in Figure 1 (22, 26).

HBOT promotes a variety of antioxidant enzymes. It induces the activation of transcription factors and the gene expression of antioxidants. It induces Nrf2, which is a redox-sensitive transcription factor that acts on genes of Hem-oxygenase-1 (HO-1), quinone oxidoreductase 1, and glutathione S-transferase, reducing the ROS load. HO-1 is also known as HSP-32 and has a neuroprotective effect. HBOT protection is also enhanced by increasing the levels of glutathione, superoxide dismutase (SOD), catalase glutathione peroxidase (GPx), and reductase (GR) (68, 71–73). Both GPx and GR are significantly increased with HBOT and are negatively correlated with infarct volume (*p* < 0.01 and *p* < 0.05, respectively) (Figure 1) (73, 74).

HBOT has also shown beneficial effects on acute (75–77), subacute (78), and chronic stroke (79). There is still no good evidence to suggest that HBOT should be the gold standard for managing acute stroke, but its clinical benefits cannot be entirely ruled out. New research studies show that HBOT reduces and improves functional symptoms, improves mobility, and reduces treatment time for patients (80).

## HBOT in neonatal HIE and IRI

The first cases of hypoxic ischemic encephalopathy (HIE) treated with hyperbaric oxygen therapy (HBOT) were reported in the first half of the 1960s (81, 82). This practice continued in the former USSR and was reported in the early 1980s, with more than 1,400 cases treated (83–85). The use of HBOT in neonatal HIE was discontinued for a long period due to technical factors, logistics between the neonatal and HBO departments, little experience in pediatric and neonatal patients, and fear of side effects.

Further publications were reported in the early 2000s from Mexico and China (86–88). The Mexican group recommended not treating patients under 34.5 weeks of pregnancy and < 1.2 kg of weight due to the higher lung and eye complications related to prematurity. They also published the proposed HBOT protocol for neonatal HIE (86). Neonatal HIE is more frequently encountered in neonates with an Apgar score under 3 at 1 min and 5 at 5 min, with a pH lower than 7.2, and a resuscitation time longer than 8 min. Neonates presenting with these conditions will develop cerebral edema at 4 h and convulsions at 6 h post-delivery. Due to the severity of this condition, neonates require NICU care that must be continued during HBOT (86).

Similar to other ischemic/hypoxic conditions, the advantages of HBOT in HIE in neonates are time dependent. HBOT should be initiated as early as possible, preferably within the first 4 h, but ideally during the 1 h after delivery. Furthermore, the treatment pressure should be between 1.5 and 1.8 ATA with a duration of 45 to 60 min to reduce possible HBOT side effects. Only one treatment is needed when HBOT is applied within the first 4 h after delivery (86).

A systematic review conducted in China reported that HBOT reduced mortality and neurologic sequelae in term neonates with HIE. Their protocol involved using HBOT at 1.5–1.7ATA for 60 to 90 min, one to three times a day. HBOT was administered within 24 h in most cases, but the exact time to administer it was not specified. The results suggested that HBOT may reduce mortality (OR 0.26, CI 95% 0.14 to 0.46) and neurologic sequelae (OR 0.41, IC 95% 0.27 to 0.61). According to the authors, the reports were of poor quality and suggested the need for adequately powered, high-quality, randomized controlled trials (88).

In another article published in Chinese, they presented their experience with 60 patients treated with three different treatment pressures (1.4, 1.5, and 1.6 ATA) for 60 min, once a day for 7 days. They measured serum levels of malondialdehyde (MDA), SOD, NO, and NOS before and after HBOT. Serum SOD levels increased, while serum levels of MDA, NO, and NOS decreased (*p* < 0.05). The neonatal behavioral and neurological assessment (NBNA) scores in the three groups increased significantly after HBOT (*p* < 0.05). There were no side effects reported (89).

A meta-analysis was conducted in China with 46 clinical RCTs that included 4,199 patients with neonatal HIE treated with HBOT. Their results indicated that HBOT significantly improved the total efficiency (TEF) of treatment in neonates with HIE (OR 4.61, 95%CI 3.70 to 5.75 – *p* < 0.0001), reduced the risk of sequelae (OR −0.3, 95%CI 0.16 to 0.33, *p* < 0.0001), and increased the NBNA scores (MD −4.51, 95%CI 3.83 to 5.19, *p* < 0.0001). They concluded that HBOT is a potential complementary treatment for neonatal HIE, but the study protocols had great heterogeneity (89). There was no information on the time to start HBOT after delivery, and the range of treatments was 5 to 90, at pressures ranging from 1.4 to 1.6 ATA once a day (88).

In another Chinese article involving 80 patients with neonatal HIE, HBOT was associated with monosialotetrahexosylganglioside sodium (GM1), a neurotrophic factor extracted from the porcine brain. The patient was > 2.5 kg, and gestational age ranged from 37 to 41 weeks. All the patients were treated within 12 h of birth. The treatment pressure was kept between 1.3 and 1.5 ATA with 80% oxygen for 20 to 25 min, once a day in a 10-day cycle, 1 week apart, and three cycles in total. They concluded that GM1 combined with HBOT can significantly improve both short-term and long-term nervous system development and brain physiology in children with moderate and severe HIE (90). Despite their good results, the oxygen treatment does not fulfill the definition of hyperbaric oxygen due to the treatment pressure (1.3 ATA), treatment time of 20–25 min, and the use of oxygen at 80%.

## HBOT side effects in neonatal HIE

In general, term neonates have good antioxidant defenses. Neonates have the highest antioxidant defenses encountered in life in preparation for breathing oxygen at birth. Nevertheless, it is not the same for premature babies, especially those under 34.5 weeks of gestation (86). It has been reported that HBOT might prevent the retinopathy or retrolental fibroplasia of premature babies (91–93). In the articles reviewed, there were few side effects of HBOT in neonatal IHE. It is probably related to the reduction of IRI and the low treatment pressure used. In the event of pulmonary oxygen toxicity, pulmonary surfactant should be readily available to adequately and promptly manage it. A bi-spectral index monitor (BIS) could be used to monitor the EEG during the treatment to monitor probable central nervous system (CNS) poisoning. Retinopathy does not appear to be a problem unless the neonate is under 34.5 weeks of gestation.

## Other treatments for neonatal HIE

A systematic meta-analysis of 11 randomized controlled studies (RCTs) published in 2013 investigated the effects of selective head cooling and whole-body cooling initiated within 6 h of birth in infants with a gestational age of > 35 weeks and moderate to severe HIE. The analysis found that hypothermia was associated with a reduced risk of death or major neurodevelopmental disability by 18 months of age (RR −0.75, 95%CI 0.68 to 0.83). Long-term follow-up of these studies is still pending (94, 95). Current protocols for hypothermia are only partially effective, with improved outcomes if started within the first 6 h of birth. Despite the beneficial effects of hypothermia, 48% experience devastating complications. Various pharmacological treatments (erythropoietin, allopurinol, melatonin, cannabinol, and exedin-4/exenatide) have been examined for use in combination with hypothermia. However, there is a need for more studies to determine their efficacy (96). Recently, a Chinese article proposed the combination of HBOT and a mild hypothermic mattress, suggesting potential synergistic benefits (97).

## Conclusion

Almost all life on Earth depends on oxygen and has to adapt to an oxygen-rich environment. The appearance of antioxidants, chloroplasts, and mitochondria enabled life on Earth. It flourished into the five different lines that inhabit it now. Oxygen has become one of the fundamental components of cell-to-cell communication and signaling through its reactive oxygen species. Many of the cellular responses during normal situations, but especially during hypoxia and reperfusion injury, depend on the cellular redox balance. It governs most of the acute responses during cellular stress and participates in the maintenance of homeostasis and allostasis. Hyperbaric oxygen therapy helps restore oxygen partial pressure at the cellular and tissue levels, supports cell communication and signaling, and maintains the redox balance, especially in critical situations such as hypoxic ischemic encephalopathies and ischemic reperfusion injury.

HBOT has not been systematically used for neonatal HIE due to concerns about treating pediatric and neonatal patients. The possible side effects of HBOT in neonates have hindered its use. The treatment should be applied ideally within the first hour after birth and requires close coordination between the neonatal and hyperbaric medicine departments. It is recommended that a neonatologist be on the hyperbaric team to maintain the same quality of care in the hyperbaric unit as in the neonatal ICU. The hyperbaric team must be trained in the management of neonates, and the neonatal team must know the particularities of HBOT in neonates.

However, there are technical and equipment issues. There are no neonatal IV pumps or ventilators. Thus, to provide IV fluids, we need to turn the pump on and off to guarantee the appropriate volume that neonates need. Since there are no hyperbaric neonatal ventilators, a neonatologist must ventilate the patient with an Ambu bag inside the chamber. To avoid neonatal hypothermia during HBOT, the bed linen should be preheated at 40°C in a vapor autoclave.

Selective head or total body cooling is a standard treatment but must be optimized. It is part of the pathophysiology of HIE, but not all of it. HBOT has a greater impact on hypoxia and ischemia-reperfusion injury than hypothermia in neonatal HIE. It is true that not all hospitals have a hyperbaric chamber, but even in those with hyperbaric departments that treat acute IRI, patients do not get referred for treatment.

There is a need to expedite treatments to meet the window of opportunity of < 6 h, ideally within the first hour after delivery. It could be accomplished if the OBGYN, Neonatal, and Hyperbaric Departments worked together as a real multidisciplinary team. There are many challenges, but it has been proven that a neonate can be treated in the hyperbaric chamber within 30 min of delivery (86).

Since selective head and total body cooling could be improved, it would be favorable to combine both treatments. First, physicians should start with HBOT very early (< 1–4 h). When administered during this time window, the Mexican experience showed that only one treatment was needed to reverse HIE. Then, hypothermia could be used to complement and continue the treatment. The patients should be evaluated daily to establish the duration of the hypothermia treatment. Two very strong paradigms can make this happen. First, physicians should change their mindset regarding the acute application of HBOT in HIE and IRI. HBOT is used for several acute ischemic conditions but has not been extended to neonatal HIE. Second, the mindset of neonatologists should be changed to apply HBOT very early and then continue with hypothermia.

Neonatal HIE is a devastating injury that has one of the largest health inequities and carries a large global burden of disease (GBD), especially in moderate and severe cases. Despite efforts, the real morbidity, mortality, and lethality of neonatal HIE have not shown substantial reductions over the past 20 years. Thus, it would be interesting to incorporate other treatments to improve the outcomes of neonatal HIE, not only pharmacological but also HBOT.

Currently, neonatal HIE is not an accepted condition by the Undersea and Hyperbaric Medical Society (UHMS), although other acute ischemic conditions are. This lack of recognition means that medical insurance companies do not reimburse for neonatal HIE, further hindering its use. Finally, the basic science supports the potential benefits of HBOT in treating neonatal HIE, but it is important to develop more clinical trials (RCT) to show its real value and take it from the lab to clinical practice.

## Author contributions

ES-R: Conceptualization, Formal analysis, Investigation, Supervision, Visualization, Writing – original draft, Writing – review & editing. VL: Writing – original draft, Writing – review & editing.
